# Transfer of Extracellular Vesicle-Associated-RNAs Induces Drug Resistance in ALK-Translocated Lung Adenocarcinoma

**DOI:** 10.3390/cancers11010104

**Published:** 2019-01-17

**Authors:** Hoi-Hin Kwok, Ziyu Ning, Peony Wing-Chi Chong, Thomas Shek-Kong Wan, Margaret Heung-Ling Ng, Gloria Y.F. Ho, Mary Sau-Man Ip, David Chi-Leung Lam

**Affiliations:** 1 Department of Medicine, Li Ka Shing Faculty of Medicine, The University of Hong Kong, Hong Kong, China; kwokh@hku.hk (H.-H.K.); chloening2014@hotmail.com (Z.N.); peonyki@hku.hk (P.W.-C.C.); msmip@hku.hk (M.S.-M.I.); 2 Department of Anatomical and Cellular Pathology, Faculty of Medicine, The Chinese University of Hong Kong, Hong Kong, China; wantsk@hku.hk (T.S.-K.W.); margaretng@cuhk.edu.hk (M.H.-L.N.); 3 Department of Occupational Medicine, Epidemiology & Prevention, Feinstein Institute for Medical Research, Hofstra Northwell School of Medicine, Great Neck, New York, NY 11021, USA; gloria_ho4@hotmail.com

**Keywords:** extracellular vesicles, TKI-resistance, anaplastic lymphoma kinase, non-small cell lung cancer, intratumoural heterogeneity

## Abstract

Anaplastic lymphoma kinase (*ALK*) translocation is an actionable mutation in lung adenocarcinoma. Nonetheless tumour consists of heterogeneous cell subpopulations with diverse phenotypes and genotypes, and cancer cells can actively release extracellular vesicles (EVs) to modulate the phenotype of other cells in the tumour microenvironment. We hypothesized that EVs derived from a drug-resistant subpopulation of cells could induce drug resistance in recipient cells. We have established *ALK*-translocated lung adenocarcinoma cell lines and subclones. The subclones have been characterized and the expression of EV-RNAs determined by quantitative polymerase chain reaction. The effects of EV transfer on drug resistance were examined in vitro. Serum EV-RNA was assayed serially in two patients prescribed ALK-tyrosine kinase inhibitor (ALK-TKI) treatment. We demonstrated that the EVs from an ALK-TKI-resistant subclone could induce drug resistance in the originally sensitive subclone. EV-RNA profiling revealed that miRNAs *miR-21-5p* and *miR-486-3p*, and lncRNAs *MEG3* and *XIST* were differentially expressed in the EVs secreted by the resistant subclones. These circulating EV-RNA levels have been found to correlate with disease progression of *EML4-ALK*-translocated lung adenocarcinoma in patients prescribed ALK-TKI treatment. The results from this study suggest that EVs released by a drug-resistant subpopulation can induce drug resistance in other subpopulations and may sustain intratumoural heterogeneity.

## 1. Introduction

Rearrangement of chromosome 2 leads to fusion of the echinoderm microtubule-associated protein-like 4 (*EML4*) and anaplastic lymphoma kinase (*ALK*) gene to form the constitutively active *EML4-ALK* oncoprotein. Although *EML4-ALK* fusion occurs in only 3–5% of lung adenocarcinomas [1], it is a therapeutic target in non-small cell lung cancer (NSCLC).

The first-generation ALK-tyrosine kinase inhibitor (TKI) crizotinib is a small molecule TKI of *ALK*, *MET* and *ROS1* kinases. Crizotinib was previously the recommended first-line treatment for *ALK*-translocated NSCLC. Although most cases of *ALK*-translocated NSCLC respond initially to crizotinib, disease progression occurs approximately one year after treatment. The resistance mechanisms of crizotinib can be classified as acquisition of secondary mutations in the *ALK* kinase domain, *ALK* fusion gene amplification, or activation of bypass signaling pathways [2]. Ceritinib and alectinib are second-generation *ALK* inhibitors with improved potency and selectivity against the kinase activities of *ALK*. Due to its proven superior efficacy and lower toxicity, Alectinib is now approved as first-line treatment for *ALK* NSCLC and in patients prescribed crizotinib in whom disease progression has occurred. 

Intratumoural heterogeneity (ITH) has been recognized in all types of cancer. The subpopulations of tumour cells with diverse phenotypes and genotypes contribute to treatment resistance and metastasis in lung cancer [3]. Dynamic interactions between subpopulations of tumour cells and stromal cells within the tumour microenvironment are believed to be critical for tumour maintenance, and may also drive the development of drug resistance. Blocking of relevant inter-cellular communications may create a therapeutic window for overcoming drug resistance [4].

Extracellular vesicles (EVs) include exosomes, microvesicles, and apoptotic bodies. Exosomes, in particular those with 30–150 nm diameter, are secreted by most cell types into bodily fluids including blood, urine and cerebrospinal fluid, as well as in supernatants from cultured cells [5]. Tumour-derived EVs that contain biomolecules (i.e., proteins, DNA and RNA) can mediate communications between different subpopulations of cells within a tumour or between cells at distant metastatic sites. These paracrine and endocrine functions of EVs have been implicated in modulation of the tumour microenvironment [6] and creation of pre-metastatic niches at distant sites [7,8]. EVs are comprised of a phospholipid bilayer that preserves and stabilizes different types of RNA (e.g., messenger RNA [mRNA], long non-coding RNA [lncRNA] and microRNA [miRNA]) [9,10]. Analysis of cancer-derived EV-associated RNA contents can enable decryption of the biological messages released from cancer cells. Recent studies have demonstrated that cancer-derived EV-RNAs can also serve as novel circulating diagnostic or prognostic biomarkers for lung cancers [11]. Furthermore, engineered EVs that contain short interfering RNA have been shown to facilitate oncogene-targeted therapy in cancer [12].

The aims of this study were: (1) To establish subclones of *ALK*-translocated NSCLC cell lines with known drug sensitivity and resistance to crizotinib or ceritinib; (2) To identify the EV-associated RNAs involved in transmission of drug resistance and migration capability from ALK-TKI-resistant subclones to drug-sensitive subclones; (3) To determine whether these EV-associated RNAs involved in drug resistance correlate with treatment response in patients with *ALK*–translocated lung adenocarcinoma.

## 2. Results

### 2.1. Characterization of EML4-ALK Lung Adenocarcinoma Cell Line Subclones

**Figure 1:**
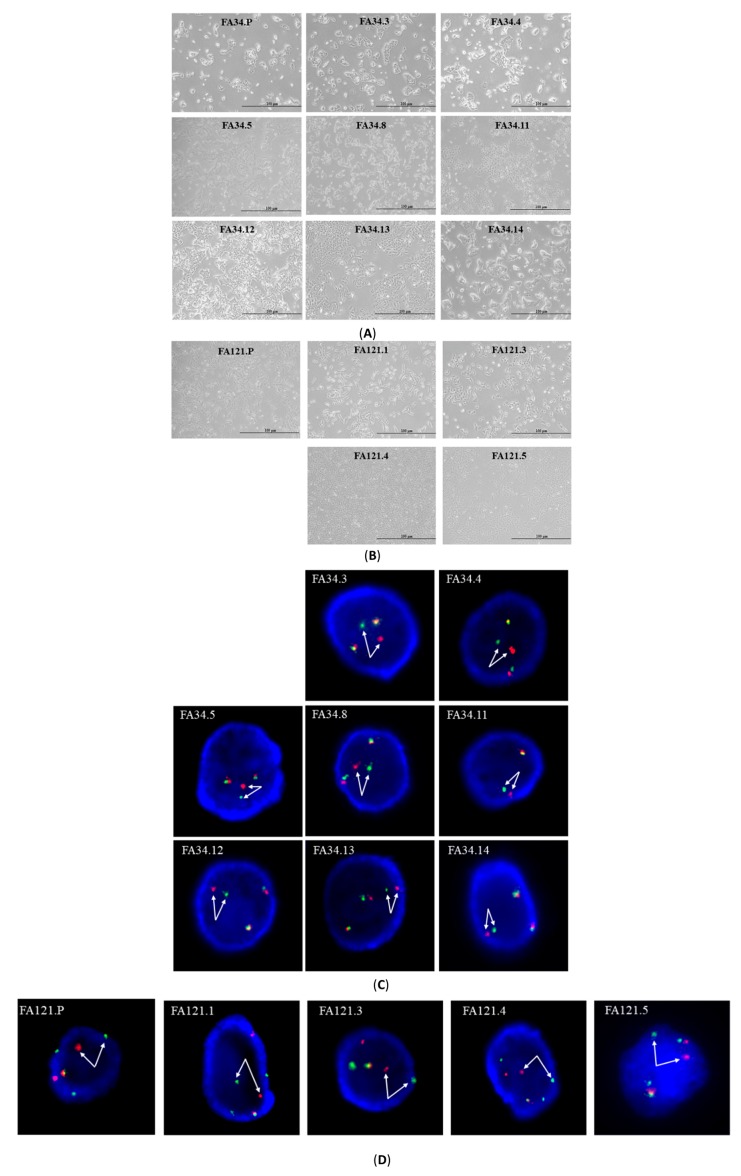

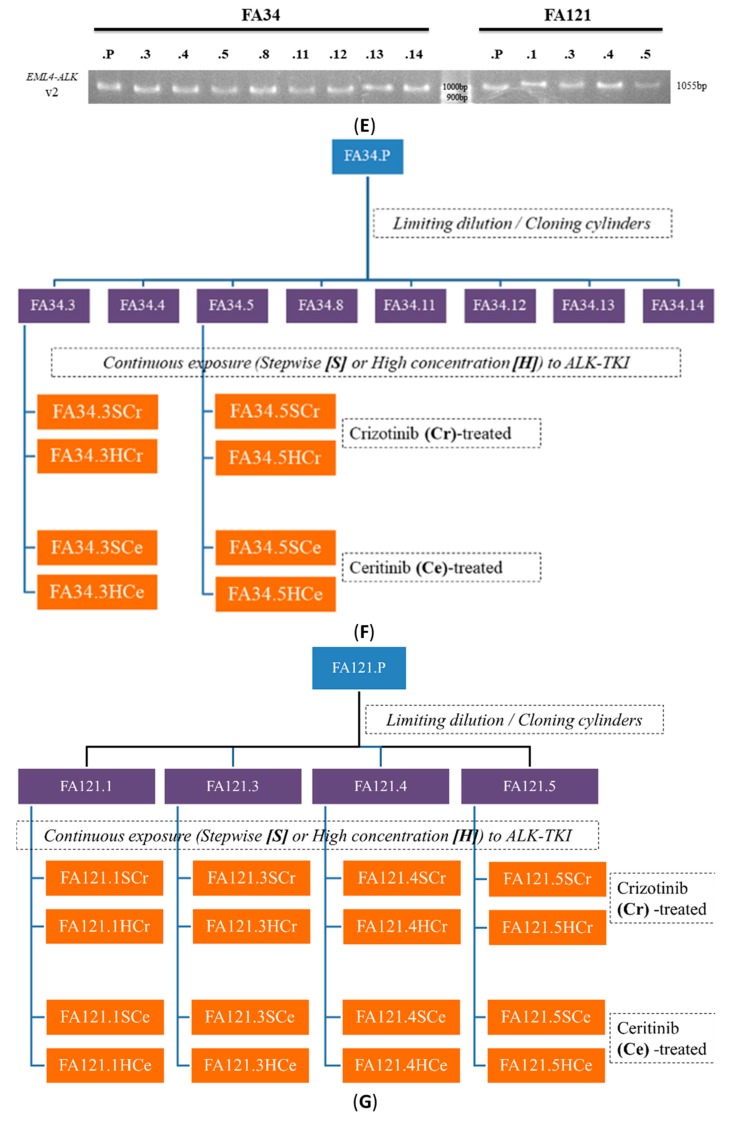
Characterization of the two echinoderm microtubule-associated protein-like 4 - anaplastic lymphoma kinase (*EML4-ALK)*-translocated lung adenocarcinoma cell lines and their subclones. The morphology of the various subclones derived from two parental lines (**A**) FA34.P and its subclones, and (**B**) FA121.P and its subclones. Split of *ALK* gene in (**C**) FA34 and (**D**) FA121 parental lines and their subclones were validated by ALK-specific break-apart fluorescence *in situ* hybridization (FISH) probe (arrows). (**E**) *EML4-ALK* variant 2 was reconfirmed by reverse transcription-polymerase chain reaction (RT-PCR) in all FA34 and FA121 parental lines and their subclones. Generation of crizotinib- or ceritinib-resistant *EML4-ALK* lung adenocarcinoma cell lines (**F**) FA34 and (**G**) FA121. Crizotinib- (Cr) or ceritinib (Ce)-resistant subclones derived from prolonged stepwise (S) or high concentration (H) treatment on (A) FA34 subclones or (B) FA121 subclones.

Subclones (Figure 1A,B) were established respectively by single cell cloning or cloning cylinders from each of the parental lung adenocarcinoma cell lines, FA34 and FA121. *ALK* break apart FISH assays were used to confirm *ALK* chromosome rearrangement in all parental and subclone cell lines. Consistent chromosome rearrangements were detected in all FA34 (Figure 1C) and FA121 (Figure 1D) cell lines and their respective subclones. PCR products with size 1055 bp were obtained from all the cell lines and subclones (Figure 1E); this confirmed that all has variant 2 of *EML4-ALK* rearrangement (i.e., fusion at exon 20 of *EML4* with exon 20 of *ALK*). The sensitivity of these cell lines against the three ALK-tyrosine kinase inhibitors (TKI), namely crizotinib, ceritinib and alectinib, was examined (Table 1). The potency of the two second-generation ALK-TKIs, ceritinib and alectinib, was higher than that of the first-generation ALK-TKI crizotinib in all the subclones. Different subclones derived from the same cell line showed variations in sensitivity to the same ALK-TKIs (Table 1A). 

### 2.2. Establishment of Drug Resistant Lung Adenocarcinoma Cell Lines and Determination of Relevant Drug Resistant Mechanisms

All subclones of the two *ALK* cell lines (FA34 and FA121) were continuously incubated with crizotinib (Table 1B) or ceritinib (Table 1C) with either stepwise escalation of drug concentrations or high bolus concentrations. For FA34 subclones (Figure 1F), only FA34.3 and FA34.5 survived following continuous incubation with crizotinib. The IC_50_ of FA34.3 against crizotinib increased from 0.4 μM to approximately 20 μM (a 50-fold increase), while the IC_50_ of FA34.5 increased from 2 μM to 20 μM (a 10-fold increase) in the resistant subclones (named FA34.3SCr, FA34.3HCr, FA34.5SCr and FA34.5HCr, respectively).

All four subclones of FA121 (Figure 1G) survived after prolonged incubation with crizotinib, and their IC_50_ against crizotinib increased from 2-fold to 130-fold. Continuous ceritinib exposure eliminated most of the FA34 subclones but not those of FA34.3 and FA34.5 (named FA34.3SCe, FA34.3HCe, FA34.5SCe and FA34HCe, respectively). The IC_50_ of resistant FA34.5 increased from 0.4 μM to 5 μM or 2 μM in two treatment methods. All the FA121 subclones survived after prolonged crizotinib or ceritinib treatment. These results also suggested that different levels of intratumoural heterogeneity among patients may be associated with different clinical outcomes during ALK-TKI treatment.

Direct sequencing of the cDNA amplicon of the *ALK* kinase domain revealed a resistant mutation C1156S in FA121.3HCr; IC_50_ of the recipient cells against crizotinib increased from 0.1 μM to approximately 13 μM (Table 1B). Another resistant mutation T1151M was found in several ceritinib-treated cell lines including FA121.3SCe and FA121.4SCe, and also in FA121.4HCe (Table 1C). *ALK* gene copy number gain (CNG) representing *ALK* gene amplifications was found in all FA34-resistant subclones regardless of treatment with crizotinib or ceritinib. Some FA121-resistant subclones also acquired *ALK*-CNG after treatment with crizotinib or ceritinib.

### 2.3. Identification of ALK-TKI Resistance-Related EV-RNAs in Lung Adenocarcinoma Subclones

**Figure 2:**
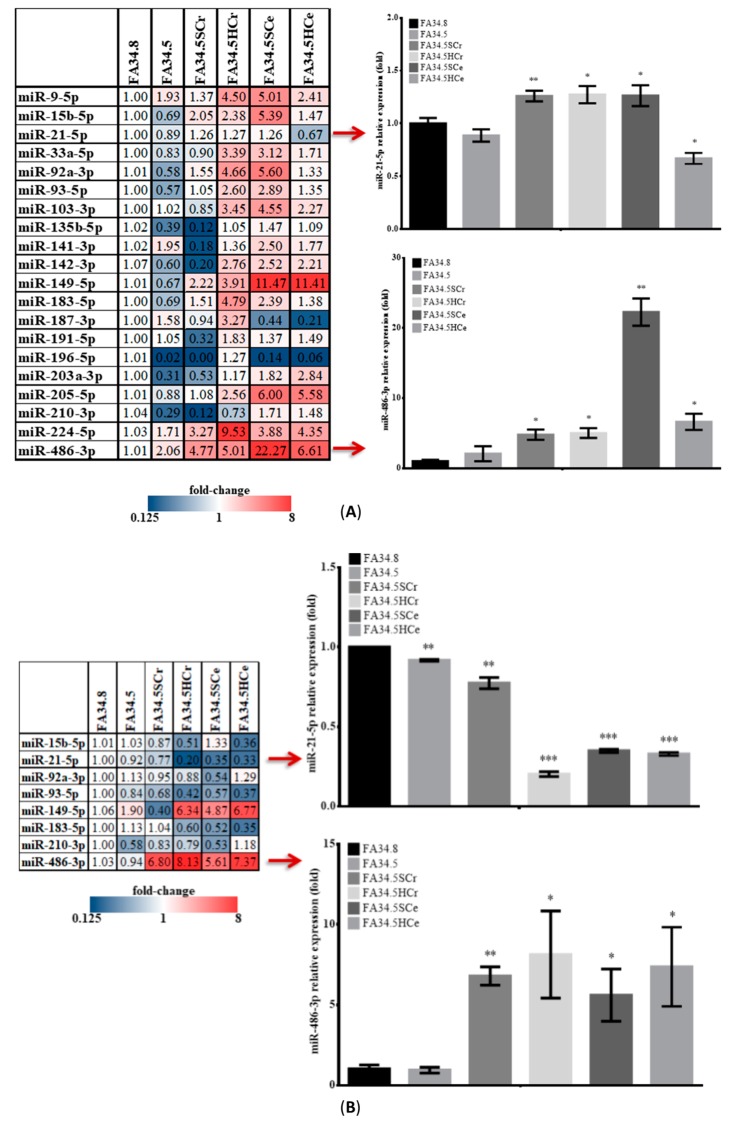

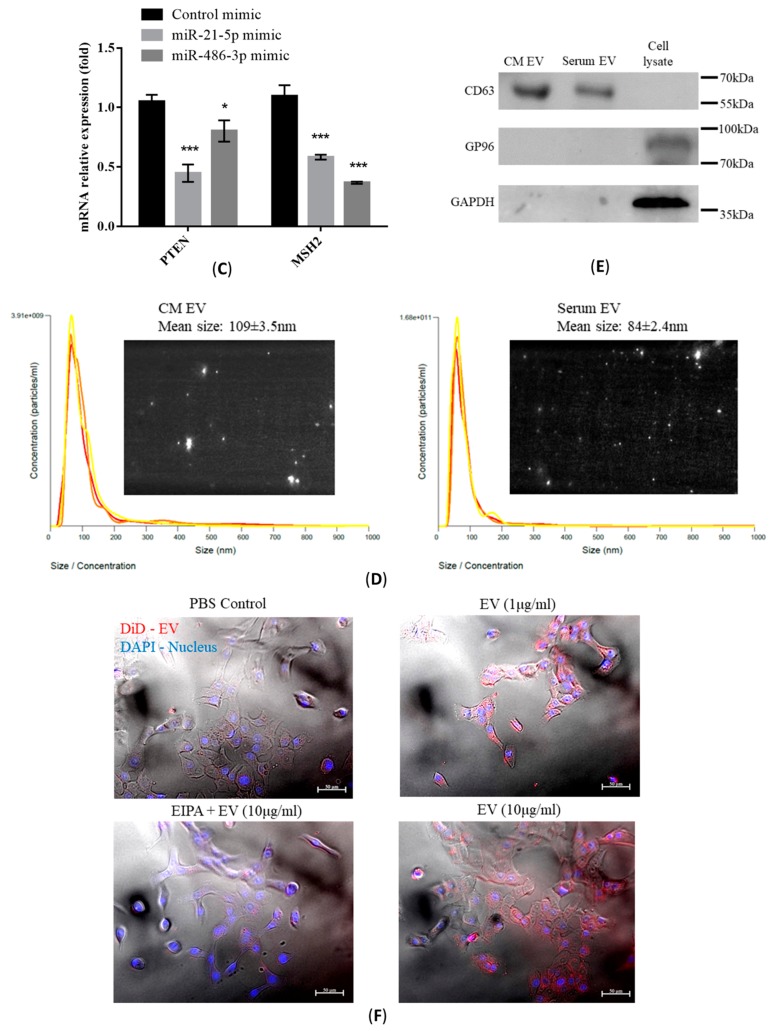
Profiles of intracellular and EV-associated miRNAs in FA34 resistant subclones. The (**A**) intracellular and (**B**) EV expression level of a panel of selected cancer-related miRNAs in the ALK-TKI-sensitive subclone FA34.8 and also the resistant subclones derived from FA34.5. The values are presented as mean ± S.D. from independent triplicate experiments. * *p* < 0.05, ** *p* < 0.01 vs FA34.8. (**C**) The relative expression of *PTEN* and *MSH2* mRNA in FA34.8 transfected with *miR-21-5p* mimic or *miR-486-3p* mimic (1 nM) for 24 h. The values are presented as mean ± S.D. from independent triplicate experiments. * *p* < 0.05, *** *p* < 0.001 vs control mimic. (**D**) Characterization of EVs. Particle size analysis of EVs isolated from conditioned medium (CM) from FA34.P (left) or serum from a lung cancer patient (right). (**E**) EV protein markers CD63, and intracellular protein GP96 and GAPDH of isolated EVs from CM, serum or cell lysate were detected by immunoblot analysis with specific antibodies. (**F**) FA34.8 were incubated with DiD-labeled EVs (red) for 1 h prior to capture of fluorescent and phase contrast images (scale bar = 50 μm).

Next we compared the intracellular (Figure 2A) and EV-associated (Figure 2B) expression of miRNAs in the ALK-TKI-sensitive subclones FA34.8 and the drug-resistant subclones FA34.5. This would allow for identification of EV-miRNAs that were differentially expressed in drug-resistant cells and in the EVs they released. 

Intracellular *miR-21-5p* and *miR-486-3p* level was significantly increased in drug-resistant subclones (excepted *miR-21-5p* in FA34.5HCe) (Figure 2A). Among these 20 miRNAs, only 8 were detected in EVs released from FA34 cells. EV-*miR-486-3p* was upregulated in drug-resistant subclones (Figure 2B), but EV-*miR-21-5p* level decreased. It has been previously demonstrated that both tumour suppressor genes *PTEN* and *MSH2* can be the common regulatory targets of *miR-486-3p* [13,14] and *miR-21-5p* [15,16]. We reconfirmed that ectopic expression of *miR-486-3p* and *miR-21-5p* could repress their mRNA expression (Figure 2C).

### 2.4. Characterization of Isolated EVs

EVs were isolated from conditioned medium (CM) of FA34 or from serum samples from patients with lung cancer and prescribed ALK-TKI treatment. The average size of EVs from CM and serum was 109 nm and 84 nm respectively (Figure 2D). The purity of EVs was verified by the presence of EV protein marker CD63, and the absence of non-EV protein marker GP96 and GAPDH (Figure 2E). To confirm EV uptake, FA34.8 was incubated with isolated EVs labelled with DiD for one hour; exosome uptake in recipient cells was demonstrated by fluorescence microscopy (Figure 2F). The cells pre-treated with EIPA, a Na^+^ -H^+^ exchange inhibitor that blocks micropinocytosis, demonstrated markedly reduced exosome uptake (Figure 2F, lower left).

### 2.5. Transfer of EV-RNAs Induced Drug-Resistance and Cell Migration

**Figure 3:**
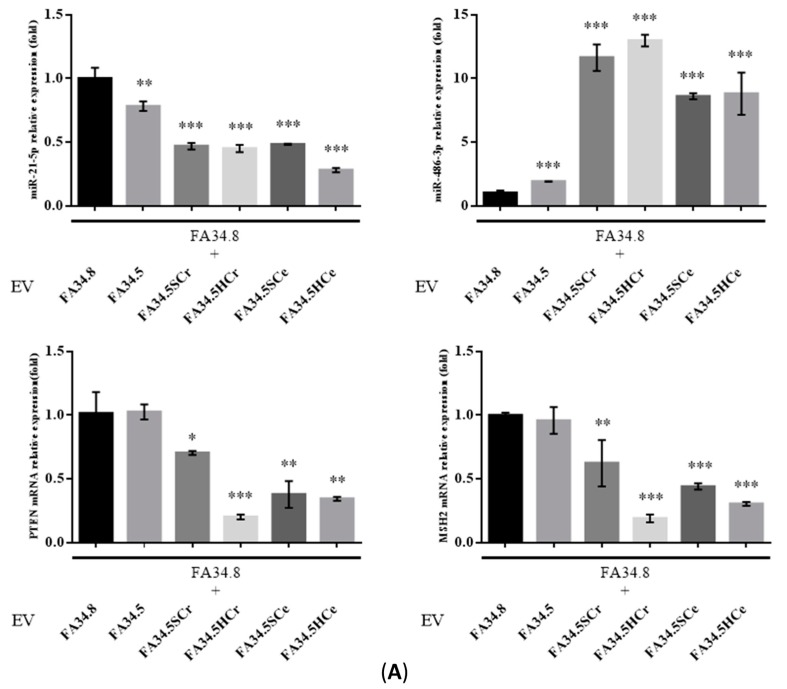

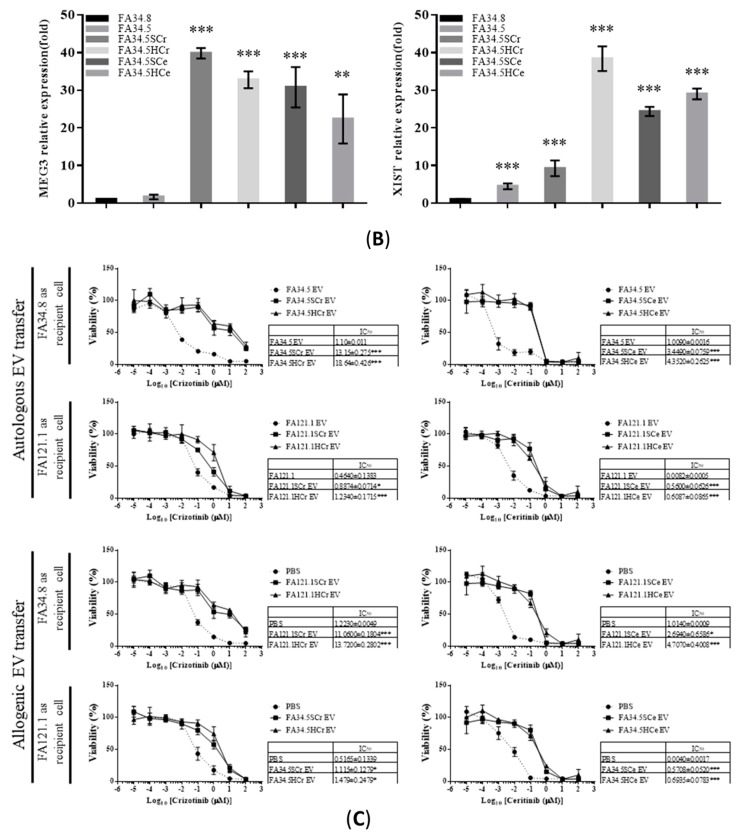
of EVs transfer from ALK-TKI resistant subclones to sensitive subclones. EVs (10 µg/mL) from FA34 or FA121 subclones were isolated and transferred to the ALK-TKI-sensitive subclones FA34.8 or FA121.1. (**A**) Relative expression of *miR-21-5p* (upper left), *miR-486-3p* (upper right), *PTEN* mRNA (lower left), and *MSH2* (lower right) was measured in FA34.8 after 24 h incubation with EVs derived from FA34-resistant subclones. (**B**) Relative expression of *MEG3* and *XIST* lncRNA in EVs isolated from different FA34 subclones. (**C**) FA34.8 or FA121.1 were pre-treated with EVs isolated from different FA34.5 or FA121.1-resistant subclones for 24 h and further treated with crizotinib or ceritinib (1 nM to 100 µM) for 72 h. The upper panel shows the results of autologous EV transfer (FA34-EV transferred to FA34 or FA121-EV transferred to FA121). The lower panel shows the results of allogeneic EV transfer (FA34-EV transferred to FA121 or FA121-EV transferred to FA34). Cell viability was determined by MTT assays. The values are presented as mean ± S.D. from independent triplicate experiments. * *p* <0.05, ** *p* < 0.01, *** *p* < 0.001 vs FA34.8 or FA121.1.

To evaluate the functional effects of EV transfer between TKI-sensitive and -resistant subclones, EVs were isolated from the TKI-resistant subclones FA34.5. These EVs were transferred to TKI-sensitive subclone FA34.8. The intracellular level of *miR-21-5p* was decreased while *miR-486-3p* in the recipient cells was increased when compared with cells treated with EVs from the same subclone FA34.8 (Figure 3A, upper panel). The expression of *PTEN* and *MSH2* was down-regulated in EV-recipient cells (Figure 3A, lower panel). Nonetheless resistant cells produced EVs containing a relatively low expression level of *miR-21-5p* that could not lead to down-regulation of intracellular level of *miR-21-5p* in the recipient cells. Other factors that could actively reduce the *miR-21-5p*, and subsequently PTEN and MSH2 in recipient cells must be present in EVs released from the resistant cells. Recent studies suggest that lncRNAs act as competitive endogenous RNA (ceRNA) of some miRNAs [17]. Two lncRNAs, *MEG3* and *XIST* have been proposed to bind to *miR-21-5p* [18,19]. We therefore determined the expression of two lncRNAs *MEG3* and *XIST* in the EVs released by different FA34 subclones. Both lncRNAs were highly expressed in the EVs of resistant subclones compared with those from the sensitive subclones (Figure 3B) indicating that these lncRNAs may be incorporated into EVs produced from resistant cells and transferred to sensitive cells, thus competitively binding with *miR-21-5p* in the recipient cells. To evaluate the functional effects of EVs transfer between subclones, the TKI-sensitive subclones FA34.8 or FA121.1 were exposed to EVs isolated from TKI-resistant subclones. Transfer of EVs produced from resistant subclones significantly increased drug resistance of sensitive subclones (Figure 3C, upper panel). To determine whether this transfer of resistance phenotype was subclone specific, we examined the effects of allogeneic EV transfer between the two *ALK*-translocated cell lines (Figure 3C, lower panel). We transferred the EVs from FA34-resistant subclone to FA121-sensitive subclone, and vice versa. The resistance-enhancing effects of these EVs were even transferrable between different cell lines. In addition, a marginal increase in cell migration was observed after TKI-sensitive cells were treated with EVs from resistant cells (Supplementary Figure S1). 

### 2.6. Prognostic Value of Circulating EV-RNAs in ALK-Translocated Lung Adenocarcinoma Patients

**Figure 4:**
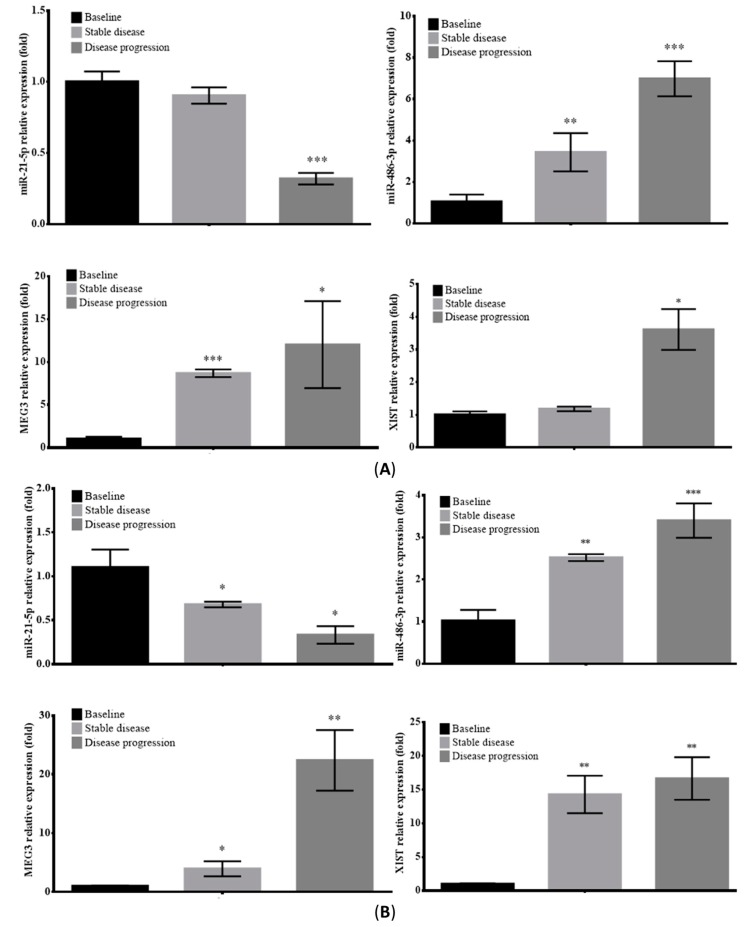
Expression changes of serum EV-associated *miR-21-5p, miR-486-3p*, *MEG3*, and *XIST* in *ALK*-translocated NSCLC patients during crizotinib or ceritinib treatment. The relative expression levels of EV-associated *miR-21-5p* (upper left), *miR-486-3p* (upper right), *MEG3* (lower left), and *XIST* (lower right) lncRNA in serial serum EV samples from *ALK*-translocated lung cancer patients treated with (**A**) crizotinib and with (**B**) ceritinib. The level of serum EV-associated miRNAs and lncRNA was determined by qPCR in the serial serum samples collected before treatment (Baseline), when disease was under control (Stable disease), and after disease progression (Disease progression). The values are presented as mean ± S.D. from technical triplicate experiments. * *p* < 0.05, ** *p* < 0.01, *** *p* < 0.001 vs. Baseline.

The expression level of circulating EV-RNAs in two *ALK*-translocated lung adenocarcinoma patients at different stages of treatment (Figure 4A,B) were assayed (complete profile in Figure S2A,B). The expression patterns of EV-RNAs were very similar in both patients. Serum EV-*miR-21-5p* showed a steady decrease during the course of treatment, through baseline, stable disease and disease progression; while EV-*miR-486-3p*, lncRNA *MEG3* and *XIST* increased throughout the course of treatment. These results suggest that these serum EV-RNAs may serve as circulating biomarkers to enable early detection of disease recurrence in patients with *ALK*-translocated lung adenocarcinoma.

## 3. Discussion

The results of this study suggest that transfer of EV-RNAs can sustain tumour subclonal heterogeneity. Although ALK inhibitors are effective in most patients with *EML4-ALK* positive NSCLC, the eventual development of acquired resistance remains a major concern in the treatment of *EML4-ALK* translocated lung adenocarcinomas. Lung tumours consist of different subpopulations of cancer cells with different phenotypic expression of cancerous properties as well as drug sensitivity [20]. Under selective pressure from chemotherapy or targeted-therapy, the interactions between subclones of cancer cells may affect treatment response and efficacy. In the lung adenocarcinoma cell line models established in this study, different FA34 subclones showed variable sensitivity to ALK-TKI, and two subclones of FA34 were able to develop resistance to ALK-TKIs. The first round of drug treatment may have introduced a selective pressure and could have suppressed the TKI-sensitive subclones (i.e., FA34.4, .8, .11, .12, .13 and .14). The surviving resistant subclones (i.e., FA34.3 and .5) could become the dominant clone after treatment. Nonetheless the results of in vitro EV transfer experiments suggest that the TKI-resistant subclones could release EV with low miR-21-5p and high miR-486-3p, and a high level of lncRNAs *MEG3* and *XIST.* The EV-RNAs then modulated TKI-sensitivity by inhibition of tumour suppressors *PTEN* and *MSH2* of the recipient subclones and enhanced their survival. This observation may in part explain the intratumoural heterogeneity of chemo-sensitivity.

In this study, we investigated the biological functions of EV-miRNAs and EV-lncRNAs. The role of *miR-21-5p* has been extensively studied in various cancers [21]. The fact that over-expression of *miR-21-5p* has been found in most tumours suggests that it may be an oncomiR that contributes to cancer cell proliferation, inhibition of apoptosis, promotion of angiogenesis [22] and metastasis [23]. The large-scale prospective clinical study BIOMILD (NCT02247453) selected 24 previously identified plasma miRNAs including *miR-21* as diagnostic signatures for lung cancer screening. A recent study using next-generation sequencing (NGS) has shown that exosomal *miR-21-5p* is downregulated rather than upregulated in lung cancer patients compared with healthy controls. These conflicting results regarding the expression of *miR-21-5p* between tissue and EVs [11,24] suggests its distinct roles in lung cancer. In our study, the contradictory expression pattern of *miR-21-5p* (high intracellular expression but low in EV) in resistant cells led us to further explore other EV-associated components, including EV-lncRNAs, that could affect the level of *miR-21-5p* in EV-recipient cells. The resistant cells produced only EVs with relatively low expression of *miR-21-5p* and could not have led to down-regulation of intracellular *miR-21-5p*, and subsequently PTEN and MSH2 in the recipient cells. The regulatory functions of lncRNAs *XIST* and *MEG3* mediated sponging of *miR-21-5p* adding another layer of complexity in subclonal communications via EVs, but also highlighted the precise control EV-cargo sorting.

For *miR-486*, both -3p and -5p strands have been proposed as oncomiR [25,26]. Intracellular *miR-486-5p* has been shown to directly suppress *NF-κB*-negative regulators and promote cancer aggressiveness through activation of *NF-κB* [25]. The prognostic power of exosomal *miR-486-5p* in NSCLC has also been demonstrated in a recent study using NGS [11]. Since a single protein can be regulated by multiple miRNAs, a cellular response such as drug resistance may involve numerous miRNAs. As cancer cells often secrete several times more EVs than normal cells [27], a panel of circulating EV-miRNA from NSCLC patients could have the potential for use as non-invasive circulating biomarkers for lung cancer patients [28].

Data from recent clinical trials show that ceritinib and alectinib, two second-generation *ALK* inhibitors, are superior to crizotinib in the first-line setting in terms of progression-free survival, especially for patients with brain metastases [29,30]. The clinical use of ALK-TKIs could be enhanced with companion biomarkers. Liquid biopsy, especially with blood sampling, may reveal the changes in oncogenic mutations along a treatment course. The resistant cell-derived EV-RNAs identified in this study may provide a source of biomarkers for monitoring of disease and treatment course. The use of serum EV-RNAs as promising biomarkers has emerged for various cancers because of their stability in circulation [10]. In contrast to free circulating miRNA, EV-miRNAs are more stable. miRNA could be packaged and enriched in EVs and then actively secreted in extracellular space for trans-cellular communication, taking part in oncogenic processes like tumour metastasis [23,31], angiogenesis [22], and chemoresistance [32]. Since specific RNA sorting [33] and cell-free miRNA biogenesis machinery [34] has been found in EVs, these mechanisms may indicate that EV-RNAs should better represent the cellular conditions than the randomly released cell-free circulating RNA [11,24].

There are some limitations in this study. First, the EV-RNA signatures, low EV-*miR-21-5p* and high *miR-486-3p*, *MEG3* and *XIST* identified in this study require clinical validation in a larger patient cohort to confirm their prognostic relevance in *ALK*-translocated lung cancer. Second, the results from this study are relevant to *ALK*-translocated lung cancer. Whether the same EV-RNA profiles are evident in other histological or molecular subtypes of lung cancer warrants further investigation. It is worth mentioning that the two cell lines used in the present study were established from malignant pleural effusions of *ALK*-TKI-naïve patients previously treated with standard platinum-based chemotherapy. Undoubtedly the EV-cargo was affected by the chemotherapy through multiple mechanisms [35]. The extent to which this could impact the predictive power of the selected exosomal miRNAs should be validated in a larger cohort. Further experiments are also warranted to explore the biological effects of EVs on relevant cancer metabolomics and epigenomic regulation of tumour heterogeneity. The novel *ALK*-translocated lung cancer cell lines together with their subclones will enable further understanding of the development of drug resistance within the heterogeneous tumour microenvironment. This will shed light on drug resistance mechanisms in *ALK*-translocated lung cancer. 

## 4. Materials and Methods

### 4.1. ALK-Translocated Lung Adenocarcinoma Cell Lines

All clinical specimens were collected after informed written consents had been given by the respective subjects. The study was approved by the University of Hong Kong/Hong Kong Hospital Authority Hong Kong West Cluster Institutional Review Board/Ethics Committee (UW 11–326).

The *EML4-ALK* lung adenocarcinoma cell line FA34 was established from a malignant pleural effusion of a 34 year-old female non-smoker with left lower lobe adenocarcinoma as described in a previous report [36]. Another *EML4-ALK* lung adenocarcinoma cell line FA121 was established from the pleural fluid of a 44-year-old male non-smoker with stage IV *EML4-ALK* translocated lung adenocarcinoma. Both patients had received standard platinum-based chemotherapy but had not been treated with an ALK-inhibitor prior to collection of pleural fluid.

The subclones of FA34 and FA121 were selected with single cell cloning either by serial dilution or by cloning cylinders. Eight subclones were generated from the FA34 parental cell line (FA34P) (the subclones were named FA34.3, FA34.4, FA34.5, FA34.8, FA34.11 (established by serial dilution), and FA34.12, FA34.13, and FA34.14 (established by cloning cylinders). Four subclones were generated from the FA121 parental cell line (FA121.P) (the subclones were named FA121.1, FA121.3, FA121.4, FA121.5 and they were all established by serial dilution methods). The parental and the subclones of FA34 were maintained in RPMI 1640 medium with 10% fetal bovine serum (FBS) (Gibco, Grand Island, NY, USA). The FA121 parental and subclones were maintained in ACL4 medium [37] supplemented with 10% FBS. All cell lines were cultured at 37 °C in a 5% CO_2_ incubator. All cell lines were established with more than 50 passages as an adherent monolayer. The identity and purity of all parental cell lines and the subclones were authenticated by polymorphic short tandem repeat (STR) DNA profiling assay (Genetica DNA Laboratories Inc, Burlington, NC, USA).

### 4.2. Fluorescence In Situ Hybridization (FISH)

Cultured cells were harvested and fixed onto positively-charged glass slides. ALK FISH was performed with the Vysis ALK Dual Specific Break Apart FISH Probe Kit (Abbott Molecular, Des Plaines, IL, USA) according to the manufacturer’s protocol. The images were analyzed and captured by the Isis FISH imaging system (MetaSystems, Altlussheim, Germany). 

### 4.3. Reverse Transcription-Polymerase Chain Reaction (RT-PCR)

To identify the variation of *EML4-ALK* in these newly established lung adenocarcinoma cell lines and subclones, RT-PCR was performed with primers specific to *EML4* intron 13 and *ALK* intron 19 [38]. Total RNA was extracted from cultured cells using Trizol (Invitrogen, Carlsbad, CA, USA). Complementary DNA (cDNA) was synthesized using the QuantiTect reverse transcription kit (Qiagen, Hilden, Denmark) according to the manufacturer’s instruction. The types of *EML4-ALK* fusion mRNA variants were identified by RT-PCR using the Fusion-RT-S and Fusion-RT-AS primers [38] (Table S1). PCR was performed with initial denaturation at 95 °C for 4 min, followed by 40 cycles of amplification (95 °C for 30 s, 55 °C for 30 s, and 72 °C for 2.5 min), and final extension at 72 °C for 10 min. The PCR products were resolved on 1.5% agarose gel and stained with SYBRsafe.

### 4.4. Establishment of Lung Adenocarcinoma Cell Lines with Resistance to Crizotinib or Ceritinib

To study the ALK-TKI resistant mechanisms, we established ALK-TKI-resistant cell lines. The subclones of FA34 and FA121 were incubated with crizotinib (Cr) or ceritinib (Ce) (Selleck Chemicals, Houston, TX, USA) with stepwise escalation (labelled with the suffix S) of drug concentrations (crizotinib: 0.02, 0.05, 0.2 µM; ceritinib: 0.01, 0.02, 0.04 µM), or high bolus concentrations (labelled with the suffix H) (crizotinib: 0.4 µM, ceritinib: 0.1 µM). IC50 values were determined by non-linear regression curve fitting using GraphPad Prism software 6.01. The IC_50_ of the drug-resistant cell lines against different ALK-TKIs was determined after 20 passages. The cells that survived after prolonged exposure to TKIs were considered drug-resistant cell lines. Drug-resistant cell lines treated with first-generation ALK-TKI crizotinib were annotated with suffix Cr; resistant cell lines treated with second-generation ALK-TKI ceritinib were annotated with suffix Ce.

### 4.5. Cell Viability Assay

FA34 and FA121 cells and their respective subclones were seeded onto 96-well plates overnight. After drug treatment for 72 h, the cells were incubated with (3-(4,5-dimethylthiazol-2-yl)-,5-diphenyltetrazolium bromide (MTT, USB Affymetrix, Santa Clara, CA, USA) (0.5 mg/mL). After incubation for 3 h, the medium was removed and the purple formazan product was dissolved with DMSO. Absorbance at wavelength 570 nm and 690 nm was measured using a microplate reader (CLARIOstar, BMG LABTECH, Mornington, Australia).

### 4.6. Detection of Secondary ALK Mutations or Amplifications

Secondary mutations in resistant cell lines were detected by direct sequencing of ALK kinase domain cDNA amplicon. In brief, the primers (EML4-E20-F and EML4-ALK-4193-R) (Table S1) flanking the *ALK* kinase domain coding region were used for PCR amplification and the amplicon size was 1055 bp. The resolved PCR product extracted from agarose gel was subjected to direct sequencing (Tech Dragon Ltd., Hong Kong, China) with the primer 3221-F (Table S1). *ALK* amplification was determined by quantitative PCR (qPCR) assay using QuantiFast SYBR Green PCR (Qiagen, Valencia, CA, USA) with the primers ALK-F and ALK-R (Table S1). The relative copy number ratio of *ALK* was expressed in comparison with the reference gene *LINE1* [39].

### 4.7. Extraction and Characterization of EVs Released from Lung Cancer Cells into Conditioned Medium and Serum

Serum samples from two *ALK*-translocated lung adenocarcinoma patients were centrifuged at 1500× *g* at 4 °C for 10 min to remove any cells. The supernatant was passed through 0.22 μm filters to remove contaminating cell debris and large EVs. Conditioned medium (CM) was collected from cells cultured in RPMI-1640 or ACL4 media supplemented with exosome-depleted FBS (System Biosciences, Mountain View, CA, USA) and also filtered through 0.22 μm filters prior to EV isolation. EVs in conditioned media or serum samples were isolated using miRCURY Exosome Isolation Kits (Exiqon, Vedbaek, Denmark) in accordance with the manufacturers’ protocols. The isolated EVs were characterized by particle size analysis and immunoblot analysis. The particle size distribution of extracted EVs was examined by NanoSight fluorescent nanoparticle tracking analysis (System Biosciences). The EV protein markers were confirmed by immunoblot analysis with specific anti-CD63 (1:1000, Proteintech, Rosemont, IL, USA), anti-GP96 (1:1000, Invitrogen), and anti-GAPDH (1:10000, Proteintech) antibodies. All relevant data of our experiments were submitted to the EV-TRACK knowledgebase (EV-TRACK ID: EV180001) [40].

### 4.8. EV-RNA Extraction, TaqMan microRNA Assay and Quantitative PCR (qPCR)

To elucidate the role of EV-miRNA in intratumoural heterogeneity, the expression level of 20 cancer-related miRNAs in different FA34-sensitive and -resistant subclones was assayed. The selection of this panel of cancer-related miRNAs was based on their biological relevance to different types of cancer [41,42]. Total EV-RNA was first extracted by TRIzol LS Reagent (Invitrogen). EV-miRNA quantitation was performed using TaqMan microRNA Assays (Applied Biosystems, Foster City, CA, USA). Then DNase-treated EV-RNA was converted to complementary DNA (cDNA) with specific stem-loop primers. All real-time PCR reactions were carried out in a StepOnePlus real-time PCR system (Applied Biosystems). For intracellular miRNAs, the relative expressions of the target miRNAs were normalized to the expressions of small RNA *RNU6B*. For EV-miRNAs, the relative expressions of the target miRNAs were normalized to the average threshold cycle (Ct) values of all miRNAs determined in the same sample. For quantitation of EV-lncRNAs *MEG3* and *XIST*, EV-RNA was reversely transcribed into cDNA by the QuantiNova Reverse Transcription Kit (Qiagen). The relative expression levels of the EV-lncRNAs were normalized to the expression levels of GAPDH. The relative expression levels of cellular *PTEN* and *MSH2* were quantified by the same method as mentioned above. The sequences of primers used are listed in Supplementary Table S1. 

### 4.9. EV Transfer

The effects of EV transfer from resistant subclones to sensitive subclones were assessed. EVs from the different resistant subclones were isolated and transferred to TKI sensitive subclones. Isolated EVs were labelled with 1,1′-dioctadecyl-3,3,3′,3′,-tetramethylindodicarbocyanine, 4-chlorobenzenesulfnate salt (DiD) (5 μg/mL) (Invitrogen). Cells were pre-incubated with 5-(*N*-ethyl-*N*-isopropyl)-amiloride (EIPA, 50 μM, Tocris Bioscience, Bristol, UK) for 30 min as indicated. Uptake of EVs in recipient cells was confirmed using fluorescence microscopy. To determine transfer of EV cargos, cellular RNA in recipient cells was extracted and the expression of specific miRNA and lncRNA assayed by qPCR as described above. To determine the functional effects of EV transfer, cell viability and motility were determined by MTT cell viability assay and cell migration assay respectively. Cell migration was performed using the transwell migration assay in EV recipient cells [43]. Briefly, ALK-TKI-sensitive subclones FA34.8 were treated with EVs (10 μg/mL isolated from CM of drug resistant subclones for 24 h. Treated cells were then subjected to transwell migration assay with corresponding EV loaded into the lower chamber. After further incubation for 20 h, the migrated cells were fixed and stained; the stain of the migrated cells was eluted for measurement of absorbance at 550 nm.

### 4.10. Serial Circulating EV-RNA Levels in ALK-Translocated Lung Adenocarcinoma Patients on ALK-TKI Treatment

Serial blood samples were taken from two patients with *ALK*-translocated lung adenocarcinoma along their treatment course. The first patient (Figure 4A) was a 56 year-old, male, non-smoker with a left upper lobe lung adenocarcinoma with *ALK* rearrangement confirmed by FISH on endobronchial biopsy. He had mediastinal lymphadenopathy and malignant left pleural effusion. Baseline blood was taken before commencement of crizotinib (Baseline), when the patient had stable disease on crizotinib (Stable disease), and when the patient had disease progression with enlargement of the left upper lobe mass while on crizotinib (Disease progression). The second patient (Figure 4B) was a 79 year-old, male, non-smoker with a left lower lobe lung adenocarcinoma and malignant left pleural effusion. *EML4-ALK* translocation was confirmed by endobronchial biopsy and the patient was initially treated with crizotinib but there was clinical disease progression despite three months of crizotinib. Therapy was changed to ceritinib with blood taken (Baseline). The patient showed stable disease (Stable disease) for the next eleven months before the disease progressed again (Disease progression) while on ceritinib. All blood samples were processed immediately upon collection and the respective serum portions were kept frozen at −80 °C for later analysis. Serum EV isolation, characterization and EV-RNA extraction were performed as described in previous sections. The study was approved by the Institutional Review Board/Ethics Committee of the Hong Kong University/Hong Kong Hospital Authority Hong Kong West Cluster (HKU/HA HKWC IRB/EC UW 16-104).

### 4.11. Statistical Analysis

EV-RNAs and proteins levels are reported as mean ± standard deviation. Normal distribution of quantitative data was confirmed. Between-group comparisons were performed with either Chi-square tests or Student’s *t*-tests where appropriate; multiple group comparisons were performed with one-way ANOVA followed by Tukey’s multiple comparisons post hoc. All statistical analyses were performed using GraphPad Prism software 6.01 (GraphPad Software Inc., San Diego, CA, USA). Statistical significance levels were all two-sided and were taken at *p* < 0.05. 

## 5. Conclusions

**Figure 5:**
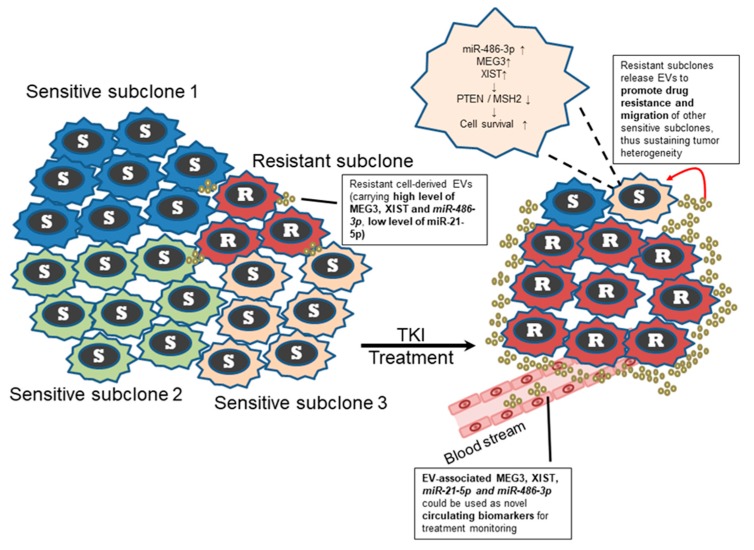
Hypothetical model for reinforcement of intratumoural heterogeneity via the transfer of EV-associated RNAs derived from resistant subclones of *ALK*-translocated lung adenocarcinoma. EV transfer from ALK-TKI resistant subclones to sensitive subclones may promote drug resistance of the sensitive subclones. Low *miR-21-5p* and high *miR-486-3p*, lncRNAs *MEG3* and *XIST* in the EVs released by the resistant subclones could inhibit the expression of tumour suppressors *PTEN* and *MSH2* by the neighboring recipient subclones and enhance their survival. Level of serum EV-associated miRNAs *miR-21-5p*, *miR-486-3p*, lncRNAs *MEG3* and *XIST* appeared to correlate with disease and treatment course in patients with *ALK*-translocated lung adenocarcinoma undergoing ALK-TKI treatment.

A panel of drug resistant subclones of *ALK*-translocated lung adenocarcinoma cell lines, namely FA34 and FA121, was established. The established subclones demonstrated a range of different drug sensitivity towards different ALK-TKIs. EV transfer from ALK-TKI-resistant subclones to sensitivity subclones showed potential mediation of drug resistance. Serum EV-*miR-21-5p*, *miR-486-3p*, *MEG3* and *XIST* levels may correlate with disease progression and treatment response in patients with ALK-translocated lung adenocarcinoma prescribed ALK-TKI treatment (Figure 5). This warrants further confirmation in a larger cohort. This study highlighted the urgent need to develop a deeper understanding of intratumoural heterogeneity via EV transfer. The results also imply that EV-RNAs may serve as a novel circulating biomarker for monitoring treatment response and progress of *ALK*-translocated lung adenocarcinoma.

## Figures and Tables

**Table 1:** IC_50_ values of different FA34 and FA121 -parental and -resistant subclones against the three ALK-TKIs tested. (**A**) The FA34 and FA121 subclones were incubated with crizotinib, ceritinib or alectinib (1 nM to 100 µM) for 72 h. Cell viability was determined by 3-(4,5-dimethylthiazol-2-yl)-2,5-diphenyltetrazolium bromide (MTT) assays. The (**B**) crizotinib or (**C**) ceritinib resistant subclones of FA34 and FA121 were treated with crizotinib, ceritinib or alectinib (1 nM to 100 µM) for 72 h. Cell viability was determined by MTT assay. The values in brackets indicate the fold-changes in IC_50_ compared with the respective subclones before prolonged TKI exposure. For secondary mutations, the *ALK* kinase domain was amplified and was sequenced to detect secondary mutations. Presence of *ALK* amplifications in these resistant subclones was determined by qRT-PCR. Wild-type (WT).

**A.** IC_50_ values of different FA34 and FA121 subclones against the three ALK-TKIs tested					
**Subclones/IC_50_ (μM)**	**Crizotinib**	**Ceritinib**	**Alectinib**		
FA34.P	0.0416	0.0535	0.0004		
FA34.3	0.4289	0.2769	0.3142		
FA34.4	0.9166	0.2484	0.0868		
FA34.5	2.306	0.4287	1.336		
FA34.8	0.2966	0.0107	0.0059		
FA34.11	0.2075	0.0212	0.0061		
FA34.12	0.42	0.3371	0.0257		
FA34.13	0.1015	0.0124	0.0004		
FA34.14	0.3062	0.0204	0.0224		
FA121.P	0.03	0.04	0.01		
FA121.1	0.3625	0.0058	0.0165		
FA121.3	0.096	0.0004	0.0009		
FA121.4	0.7736	0.7354	0.6937		
FA121.5	0.0874	0.0029	0.0067		
**B**. IC_50_ values and the resistant mechanisms of different crizotinib-resistant subclones against the three ALK-TKIs tested.					
**Subclones/IC_50_ (μM)**	**Crizotinib**	**Ceritinib**	**Alectinib**	**Secondary mutation**	**ALK amplification**
FA34.3SCr	19.6000 (471.2)	2.2790	61.8400	WT	YES
FA34.5SCr	20.1200 (8.7)	0.5629	4.9030	WT	YES
FA34.3HCr	16.8900 (39.4)	2.2560	57.0300	WT	YES
FA34.5HCr	22.9800 (10.0)	1.5690	31.4300	WT	YES
FA121.1SCr	1.2560 (3.5)	1.2760	3.0690	WT	NO
FA121.3SCr	1.0960 (11.4)	0.3091	2.0700	WT	NO
FA121.4SCr	1.9450 (2.5)	0.8955	1.8940	WT	YES
FA121.5SCr	0.1884 (2.2)	1.9920	0.3204	WT	YES
FA121.1HCr	1.6370 (4.5)	0.0293	2.0180	WT	YES
FA121.3HCr	12.860 (134.0)	1.9000	3.7180	ALK, C1156S	NO
FA121.4HCr	1.7230 (2.2)	0.0544	0.0399	WT	YES
FA121.5HCr	2.7550 (31.5)	0.0555	0.6398	WT	YES
**C.** IC_50_ values and the resistant mechanisms of different ceritinib-resistant subclones against the three ALK-TKIs tested.					
**Subclones/IC_50_ (μM)**	**Crizotinib**	**Ceritinib**	**Alectinib**	**Secondary mutation**	**ALK amplification**
FA34.5SCe	17.6900	5.5000 (12.8)	18.3600	WT	YES
FA345.HCe	24.2400	2.1280 (5.0)	11.2600	WT	YES
FA121.1SCe	0.7165	0.4356 (75.1)	0.2278	WT	NO
FA121.3SCe	0.3272	0.0557 (139.3)	0.0965	ALK, T1151M	YES
FA121.4SCe	1.4610	0.9672 (1.3)	0.1533	ALK, T1151M	NO
FA121.5SCe	1.9690	1.2190 (1.7)	0.3851	WT	NO
FA121.1HCe	1.5330	0.5631 (97.1)	10.8500	WT	YES
FA121.3HCe	7.6780	2.5650 (6412.5)	9.0510	WT	YES
FA121.4HCe	0.8824	1.1534 (1.6)	0.0845	ALK, T1151M	NO
FA121.5HCe	2.5500	0.4636 (159.8)	7.5000	WT	YES

## Supplementary Materials

The following are available online at http://www.mdpi.com/2072-6694/11/1/104/s1, Figure S1: Effects of EVs transfer from ALK-TKI resistant subclones to sensitive subclones on cell migration. Figure S2: Complete expression profiles of serum EV-associated miRNAs in two *ALK*-translocated patients. Table S1: List of primers used in this study.
