# Supplementary material for: Transfer of Extracellular Vesicle-Associated-RNAs Induces Drug Resistance in ALK-Translocated Lung Adenocarcinoma
Source: Cancers (Basel). 2019 Jan 17;11(1):104. doi: 10.3390/cancers11010104 (PMC6356387; doi:10.3390/cancers11010104)
Supplement: Supplementary file 1 [file cancers-11-00104-s001.pdf]

# Supplementary Materials: Transfer of extracellular vesicle-associated-RNAs induces drug resistance in ALK-translocated lung adenocarcinoma

**Hoi-Hin Kwok, Ziyu Ning, Peony Wing-Chi Chong, Thomas Shek-Kong Wan, Margaret Heung-Ling Ng, Gloria Y.F. Ho, Mary Sau-Man Ip and David Chi-Leung Lam**

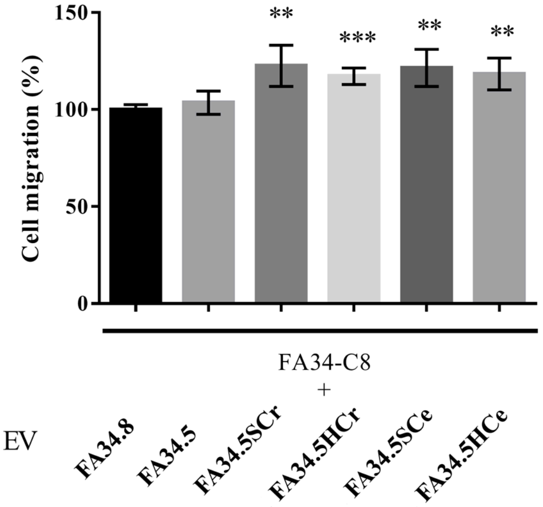


**Figure S1.** Effects of EVs transfer from ALK-TKI resistant subclones to sensitive subclones on cell migration. ** $p < 0.01$, *** $p < 0.001$ vs FA34.8.

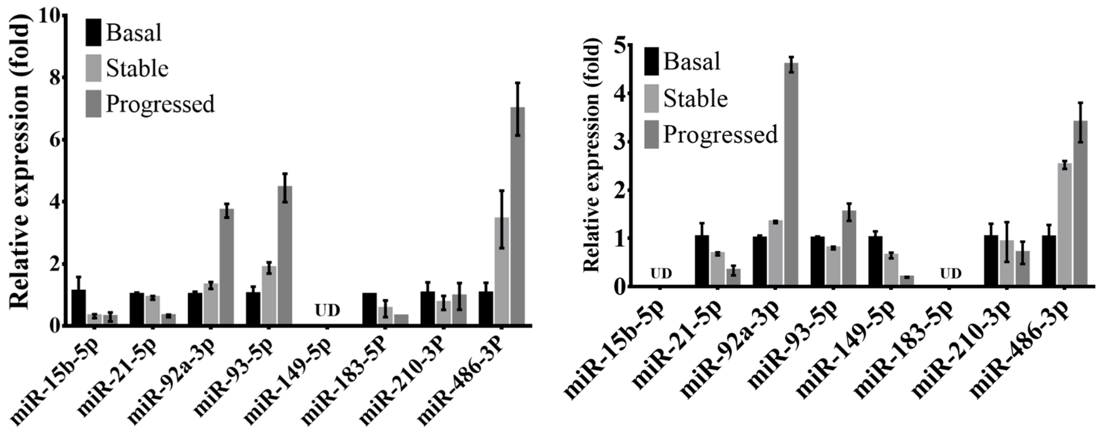


**Figure S2.** Complete expression profiles in serial serum EV samples from ALK-translocated lung cancer patients treated with (**A**) crizotinib and with (**B**) ceritinib.

**Table S1.** List of primers used in this study

| Primer | Sequence (5′–3′) |
|---|---|
| Fusion-RT-S | GTG CAG TGT TTA GCA TTC TTG GGG |
| Fusion-RT-AS | TCT TGC CAG CAA AGC AGT AGT TGG |
| EML4-E20-F | CGG GAG ACT ATG AAA TAT TGT ACT |
| EML4-ALK-4193-R | GGC AAA GCG GTG TTG ATT ACA TCC |
| 3221-F | AGA GCC CTG AGT ACA AGC TGA G |
| ALK-F | CTT TGA CTT CCC CTG TGA GC |
| ALK-R | GCA GCC TCT CCC TTA CCT C |
| LINE1-F | CCG CTC AAC TAC ATG GAA ACT G |
| LINE1-R | GCG TCC CAG AGA TTC TGG TAT G |
| MEG3-F | CTG CCC ATC TAC ACC TCA CG |
| MEG3-R | CTC TCC GCC GTC TGC GCT AGG GGC T |
| XIST-F | CTA GCT AGC TTT TGT AGT GAG CTT GCT CCT |
| XIST-R | GCT CTA GAA TGT CTC CAT CTC CAT TTT GC |
| PTEN-F | TTG GCG GTG TCA TAA TGT CT |
| PTEN-R | GCA GAA AGA CTT GAA GGC GTA |
| MSH2-F | AAT GAC TTG GAA AAG AAG ATG C |
| MSH2-R | TTA AAG AAG TCA ATT TGC TGT TG |
| GAPDH-F | GTC AGT GGT GGA CCT GAC CT |
| GAPDH-R | TGA GCT TGA CAA AGT GGT CG |

© 2019 by the authors. Licensee MDPI, Basel, Switzerland. This article is an open access article distributed under the terms and conditions of the Creative Commons Attribution (CC BY) license (http://creativecommons.org/licenses/by/4.0/).
